# Inside the Developing Brain to Understand Teen Behavior From Rat Models: Metabolic, Structural, and Functional-Connectivity Alterations Among Limbic Structures Across Three Pre-adolescent Stages

**DOI:** 10.3389/fnbeh.2018.00208

**Published:** 2018-09-24

**Authors:** Francesca Zoratto, Luisa Altabella, Naomi Tistarelli, Giovanni Laviola, Walter Adriani, Rossella Canese

**Affiliations:** ^1^Center Behavioral Sciences and Mental Health, Istituto Superiore di Sanità, Rome, Italy; ^2^Core Facilities, Istituto Superiore di Sanità, Rome, Italy; ^3^Faculty of Psychology, Università Telematica Internazionale Uninettuno, Rome, Italy

**Keywords:** adolescence, resting state fMRI, magnetic resonance spectroscopy (MRS), diffusion tensor imaging (DTI), orbitofrontal cortex, nucleus accumbens, dorsal striatum, hippocampus

## Abstract

Adolescence is an age of transition when most brain structures undergo drastic modifications, becoming progressively more interconnected and undergoing several changes from a metabolic and structural viewpoint. In the present study, three MR techniques are used in rats to investigate how metabolites, structures and patterns of connectivity do change. We focused in particular on areas belonging to the limbic system, across three post-weaning developmental stages: from “early” (PND 21–25) to “mid” (i.e., a juvenile transition, PND 28–32) and then to “late” (i.e., the adolescent transition, PND 35–39). The rs-fMRI data, with comparison between early and mid (juvenile transition) age-stage rats, highlights patterns of enhanced connectivity from both Striata to both Hippocampi and from there to (left-sided) Nucleus accumbens (NAcc) and Orbitofrontal Cortex (OFC). Also, during this week there is a maturation of pathways from right Striatum to ipsilateral NAcc, from right OFC to ipsilateral NAcc and vice versa, from left Prefrontal Cortex to ipsilateral OFC and eventually from left Striatum, NAcc and Prefrontal Cortex to contralateral OFC. After only 1 week, in late age-stage rats entering into adolescence, the first pathway mentioned above keeps on growing while other patterns appear: both NAcc are reached from contralateral Striatum, right Hippocampus from both Amygdalae, and left NAcc -further- from right Hippocampus. It's interesting to notice the fact that, independently from the age when these connections develop, Striata of both hemispheres send axons to both Hippocampi and both NAcc sides, both Hippocampi reach left NAcc and OFC and finally both NAcc sides reach right OFC. Intriguingly, the Striatum only indirectly reaches the OFC by passing through Hippocampus and NAcc. Data obtained with DTI highlight how adolescents' neurite density may be affected within sub-cortical gray matter, especially for NAcc and OFC at “late” age-stage (adolescence). Finally, levels of metabolites were investigated by 1H-MRS in the anterior part of the hippocampus: we put into evidence an increase in myo-inositol during juvenile transition and a taurine reduction plus a total choline increase during adolescent transition. In this paper, the aforementioned pattern guides the formulation of hypotheses concerning the correlation between the establishment of novel brain connections and the emergence of behavioral traits that are typical of adolescence.

## Introduction

Resting-state functional magnetic resonance imaging (rs-fMRI) is a non-invasive technique that provides critical information about the functional “*in vivo*” organization and the connectional architecture of the brain by measuring spontaneous “*in vivo*” fluctuations in the blood oxygen level dependent (BOLD) signal (Shou et al., 2014).

This technique plays an important role in revealing the sequence of functional changes during development; such information may be of interest to depict how neural correlates do change along with behavior during key developmental phases. There is a number of studies where fMRI was applied in order to achieve information about brain development across adolescence: the teen brain is characterized by enormous plasticity and common steps, with a period of synaptic overproduction followed by the loss of needless synapses. For instance, fMRI was used in addition to structural MRI in order to investigate the neural substrates of emerging social cognitive proficiency (Burnett et al., 2010); studies about the development of cognitive self-control during adolescence also used connectivity fMRI in order to explore the basic phenomena when distant regions become linked to each other (Luna et al., 2010). Neural network change was investigated in order to highlight developmental discontinuities of fundamental brain networks, such as affective and reward circuits, and how these dramatic changes reflect on teen behavior (Brenhouse and Andersen, 2011). fMRI, along with structural MRI, was also used to investigate the effects of alcohol consumption among adolescents, demonstrating how this risky behavior can entail serious damage to the developing brain (Feldstein Ewing et al., 2014).

In general terms, this powerful technique was applied in order to investigate three major themes which characterize human brain development: (1) the spread from local to distributed and integrated networks, (2) the strengthening of some specific networks, and finally (3) the interactions among networks thus achieved, which may reflect a totally new brain organization. As a rule, those networks that mediate higher cognitive processes appear the last to mature (Giedd, 2008; Fair et al., 2009; Ernst et al., 2015).

Noteworthy, preclinical literature on animal models is still devoid of such approaches. The experiment we describe in this paper is the first of this kind, since the progressive enhancement of brain connectivity at three different adolescent stages has never been investigated so far in laboratory rats. Analyses were performed using seed-based correlation analysis, a very common approach in resting-state functional connectivity (Greicius et al., 2003; Fox et al., 2005): this method starts with an a priori selection of some regions, called “seeds,” toward which it's then possible to reveal the changed patterns of connectivity. In other words, starting from extended areas labeled with a “seed,” it's possible to follow the increased flow of information coming from small tissue volumes within other structures of the brain. These functional measurements have been then integrated with a diffusion tensor imaging (DTI) study, to define structural modifications in relevant brain areas, and with a metabolic study (by quantitative ^1^H-MRS). For choice of area, we decided to direct our efforts on hippocampus, a region which is known for enhanced neurogenesis at these age-stages. We hypothesized that age-related changes in the extent of neurogenesis would be mirrored in MRS-detectable markers: the anterior part of the hippocampus includes the dentate gyrus, i.e., the region where neurogenesis is active. With the techniques depicted above, we aimed to highlight brain's evolving connectivity across development, with a focus on juvenile (pre-pubertal) to adolescent (pubertal) transitions. To this purpose, the whole developmental period ranging from weaning (PND 21) to puberty onset (PND 40; Spear, 2000) was divided in three age-stages. Fourth, fifth and sixth week of life were carefully explored in sibling subjects.

## Materials and methods

All experimental procedures were in agreement with the European Communities Council Directive (2010/63/EEC) and the Italian law. Formal license was delivered by Animal Welfare Survey Board on behalf of Italian Ministry of Health (to GL) and was active from 2010 to 2013. All efforts were made to reduce the number of animals used, to minimize animal suffering and, if available, to utilize alternatives to *in-vivo* techniques.

Wistar male rats of “early” or post-weaning (PND 21–25, *n* = 6), “mid” or juvenile (PND 28–32, *n* = 6) and “late” or adolescent (PND 35–39, *n* = 6) age-stages were considered in a period comprised between March and April 2012 (Charles River, Calco, Italy). They were offspring of an in-colony breeding, involving six dams. Three male subjects per dam were randomly assigned to be tested at one age-stage only, so that “age” could be a within-litter factor in a split-plot design; at weaning, the non-sibling subjects were housed in pairs (of the same sex) inside polycarbonate cages (42.5 × 26.6 × 18.5 cm) with sawdust bedding. They were housed in an air-conditioned room (21 ± 1°C, relative humidity 60 ± 10%), on a 12-h reversed light-dark cycle (lights off at 7.00 am). Food (Altromin-R, A. Rieper S.p.A., Vandoies, Italy) and tap water were provided *ad libitum*.

Experiments were performed on a VARIAN/Agilent Inova MRI/MRS system (Agilent, Palo Alto, CA) operating at 4.7 T provided with an actively shielded gradient system (max 120 mT/m, 11 cm bore size), by using a volume coil combined with an electronically-decoupled and receiver-only surface coil (RAPID Biomedical, Rimpar, Germany), and exploiting a set of procedures as already described (Canese et al., 2015; Zoratto et al., 2017).

Briefly, animals were anesthetized (2.0% isoflurane in oxygen 1 L/min) and left to spontaneous breathing for the entire experiment, which lasted less than 1.5 h. Body temperature was maintained at 37.0 ± 0.1°C via an integrated heating system. An MRI-compatible pulse oximeter (MouseOx, Starr Life Sciences Corp), was placed on the right posterior leg for continuous monitoring of the heart rate, breath rate, oxygen saturation (pO_2_) and pulse distension (a surrogate parameter for blood pressure) during the MRI session. MRI protocol includes coronal gradient echo and sagittal fast spin-echo anatomical images for positioning, for subsequent fMRI, DTI and MRS scans.

### Functional connectivity study

Resting state fMRI exploiting the BOLD effect was studied by using a multi slice sagittal gradient echo sequence (FLASH sequence with encoding of one k space line per loop; TR/TE = 200/5 ms, 7 slices of 2 mm thickness, FOV 25 × 25 mm^2^, 64 × 64 matrix,140 temporal points, scan time 12 s per volume, overall 30 min). This acquisition parameters allowed to obtain maps of fMRI signals at the frequency up to 0.08 Hz; therefore, maps are including the most representative frequencies of the BOLD power spectrum, which are concentrated around 0.04 Hz in cortex, hippocampus, caudate-putamen of rat brain (Kannurpatti et al., 2008). Subjects assigned to the age groups were siblings, therefore there was a low inter-individual variability; limited differences in respiratory and cardiac frequency as well as in pulse distension (a surrogate measure of blood pressure) were observed amongst the three ages (see Table 1). Therefore, these potential confounds only negligibly affected BOLD signals. Data analysis consisted of a pre-processing step (image realignment and temporal smoothing) followed by a seed-based approach with a bootstrap resampling technique. Voxel-wise correlation maps were obtained corresponding to a given seed, for the three groups of animals.

**Table 1:** Physiological parameters during functional acquisition in isoflurane anesthetised rats during the initial 4 min (“Pre”) and the final 4 min (“Post”) of the rs-fMRI acquisitions.

	**Pre**			**Post**		
	**Heart**	**Pulse**	**Breath**	**Heart**	**Pulse**	**Breath**
	**Rate (bpm)**	**Distention (mm)**	**Rate (bpm)**	**Rate (bpm)**	**Distention (mm)**	**Rate (bpm)**
Early	500 ± 30	28 ± 10	69 ± 6	500 ± 30	25 ± 3	60 ± 10
Mid	530 ± 15	25 ± 6	76 ± 4	510 ± 10	24 ± 5	71 ± 7
Late	500 ± 20	24 ± 5	77 ± 8	500 ± 20	26 ± 6	73 ± 8

#### Pre-processing

The pre-processing was based on image realignment, to reduce the artifacts due to potential animal movement (see Canese et al., 2009), and temporal smoothing, using a moving average method (span width = 5 temporal points). None of the rats had to be excluded from the analysis, since no excessive movement of the head (defined as rapid changes of signal larger than 20% during the fMRI study) was ever observed.

#### Seed-based statistical approach with a bootstrap resampling technique

First, template images were generated from the individual correlation maps, one for the “mid” and one for the “late” age-stage groups. To do this, first one representative individual was chosen; then, all the other five same-age individuals were co-registered, by a polynomial transformation (Goshtasby, 1988), and then averaged to obtain the template (see Canese et al., 2011). Then, cross correlation maps of animals of the “early” age-stage were resampled using bootstrapping (Efron and Tibshirani, 1988) to assess data reliability and reproducibility and to set a reference to identify “active” brain voxels, through a significance threshold. Bootstrap is then applied to overcome the problem of the test-retest method (Noll et al., 1997) and/or to assess significance of activation clustering (Auffermann et al., 2002). Present bootstrap resampling is an alternative, computer-intensive method that provides a strong control of the family wise control rate, which is more conservative than the false discovery rate among the combined Type I error rate (Westfall and Young, 1993). A new distribution of resampled maps for “control” animals at early age-stage was then generated (preserving the spatial information for each voxel) and the 99th percentile of the resampled dataset was computed for each voxel. This threshold has been used to differentiate active from non-active voxels in the mean template maps of the second and third week (i.e., juvenile and adolescent transitions, respectively). In other words, we seek for a distinct set of “active” voxels, in which the cross-correlation to the seed, observed in the template obtained for animals at the mid (juvenile) and late (adolescence) age-stages, is higher than the 99% percentile of the Gaussian distribution (of cross-correlations, voxels-to-seed), obtained after re-sampling the maps obtained for the animals at the early age-stage (Canese et al., 2015; Zoratto et al., 2017).

**Figure 1:**
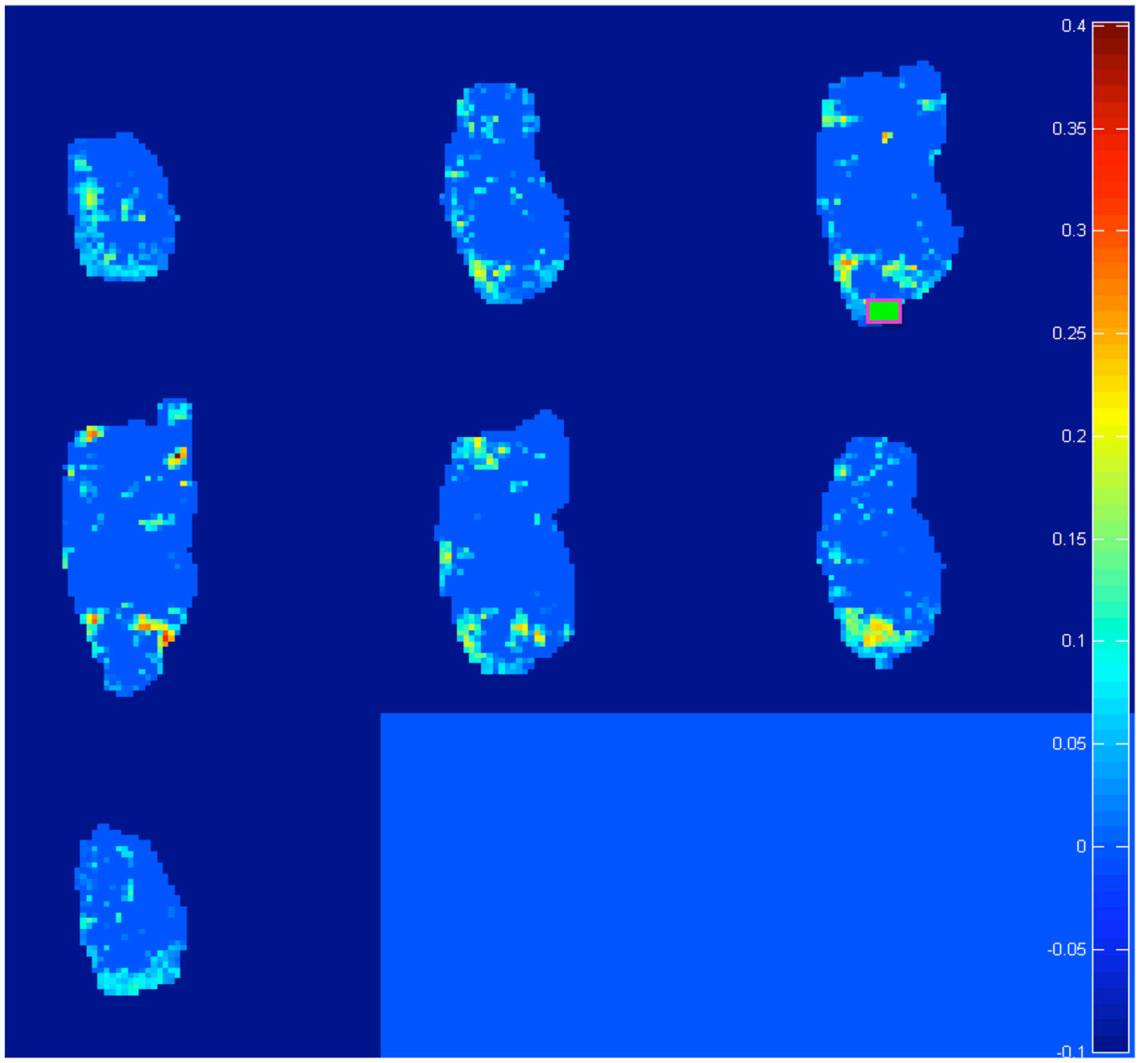
Representative example of the 20 difference maps, 10 between mid vs. early and 10 between late vs. early age-stages: active voxels out of a light blue zero are identified by the software as trespassing the threshold of the 99% percentile from the re-sampled distribution of “control” subjects at the post-weaning age-stage (see section Materials and Methods). In total, we generated and analyzed 20 such maps (10 seeds in total per two age-transition comparisons). Note: in this example, we explore “juvenile” changes of connectivity, with the seed positioned on right OFC (green square). We found increased connections (a) from contra-lateral Striatum, NAcc, Insula, and PFC (see Table 2: these targets emerge on row for OFC seed, with L → R meaning from left target to right seed) and (b) from ipsi-lateral NAcc (see Table 2: NAcc target emerging at intersection with OFC seed, with R → R). Intriguingly this last is reciprocal: for a seed on right NAcc, the ipsi-lateral OFC is detected (see Table 2: OFC target emerging at intersection with NAcc seed, with R → R). See Figure 5 for juvenile (mid vs. early) and Figure 6 for adolescence (late vs. early) transitions.

Both pre-processing and bootstrap re-sampling techniques were run using a home-made software, developed in MATLAB (Canese et al., 2015; Zoratto et al., 2017). Ten seeds were placed on brain images, located in five relevant forebrain areas and separately in both hemispheres: hippocampus (Hip), nucleus accumbens (NAcc), dorsal striatum (dStr), dorsal / medial prefrontal cortex (PFC) and orbital prefrontal cortex (OFC). For seed positioning, we (1) chose from the atlas (electronic version of Paxinos and Watson, 1998) the diagram (a 0.3-mm slice) with border of the areas, corresponding to the center of the 2-mm sagittal slice acquired by MR, (2) manually overimposed such electronic sagittal diagrams onto all MR correlation maps, (3) manually draw a 0.9–1.4 × 0.9–1.4 mm rectangle encompassing the region acting as a seed. For each seed, the cross correlation was computed between the seed's own time-course and time-courses of voxels across the rest of the brain. One representative example (with the seed positioned in right OFC, see green square) is shown in Figure 1. We considered an increase in connectivity, when comparing adolescent to juvenile transition maps, if (1) we detected an increase in the cross-correlation coefficients in some pixels of the area of interest or (2) we detected an increase in the number of active pixels (i.e., those which exceed the threshold of the 99% percentile) in that area.

### Anatomical connectivity study

**Figure 2:**
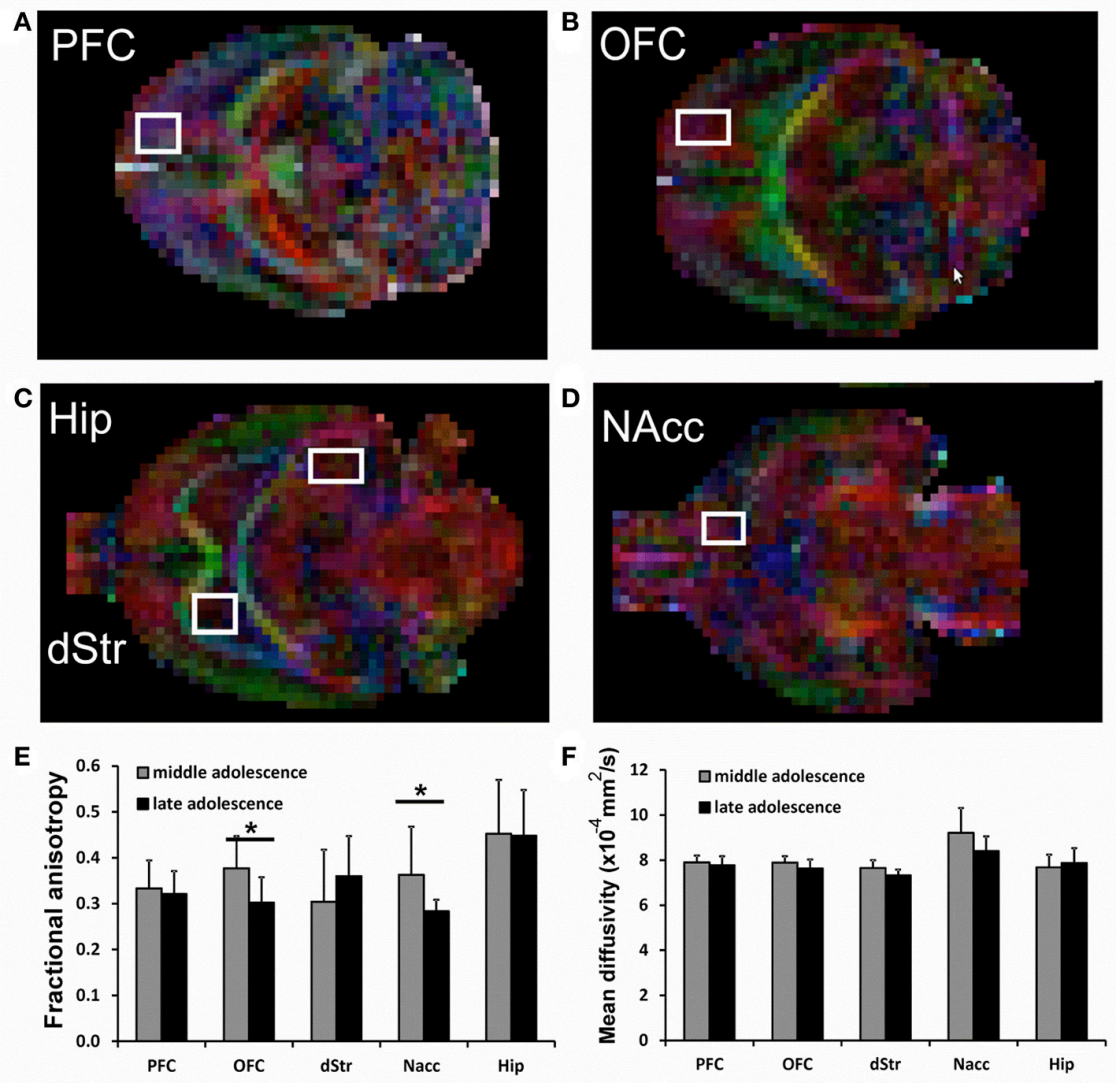
**(A–D)** representative voxels overimposed to DEC maps for the analysis of Prefrontal cortex (PFC), Orbitofrontal cortex (OFC), Hippocampus (Hip), Striatum (dStr), and Nucleus accumbens (NAcc). **(E,F)** Fractional Anisotropy (FA) and Mean Diffusivity (MD) in all analyzed brain areas. For each voxel, also the corresponding contralateral voxel was considered. Significant differences are indicated by asterisks (*p* < 0.05).

In the DTI study, a spin-echo sequence with addition of the Stejskal-Tanner diffusion gradients was applied to mid (juvenile) and late (adolescent) age-stage animals. Diffusion gradients were applied along six spatial directions. Intensity, duration and diffusion time were set to 8.27 G/cm, 8 and 25 ms respectively, given a b-value of 700 s/mm^2^. A field of view of 25 × 25 mm2 was sampled on a 64 × 64 Cartesian grid. Multi-slice diffusion tensor images were acquired (15 slices of 1 mm thickness) in the coronal plane with 2 averages and TE/TR = 50/2,000 ms. Using FSL (http://www.fmrib.ox.ac.uk/fsl/) and ImageJ (https://imagej.net) software packages, diffusivity values (fractional anisotropy, FA and mean diffusivity, MD) were derived from the tensor. The same five forebrain areas already selected for functional connectivity study were analyzed: hippocampus (Hip), nucleus accumbens (NAcc), dorsal striatum (dStr), dorsal/medial prefrontal cortex (PFC) and orbital prefrontal cortex (OFC). The analyzed regions are shown overimposed to diffusion encoded color (DEC) maps in Figure 2. FA and MD values are reposted in Figures 2E,F. Significant differences between the groups were assessed by a unpaired 2 tail *t*-test (*p* < 0.05).

### Metabolic study

**Figure 3:**
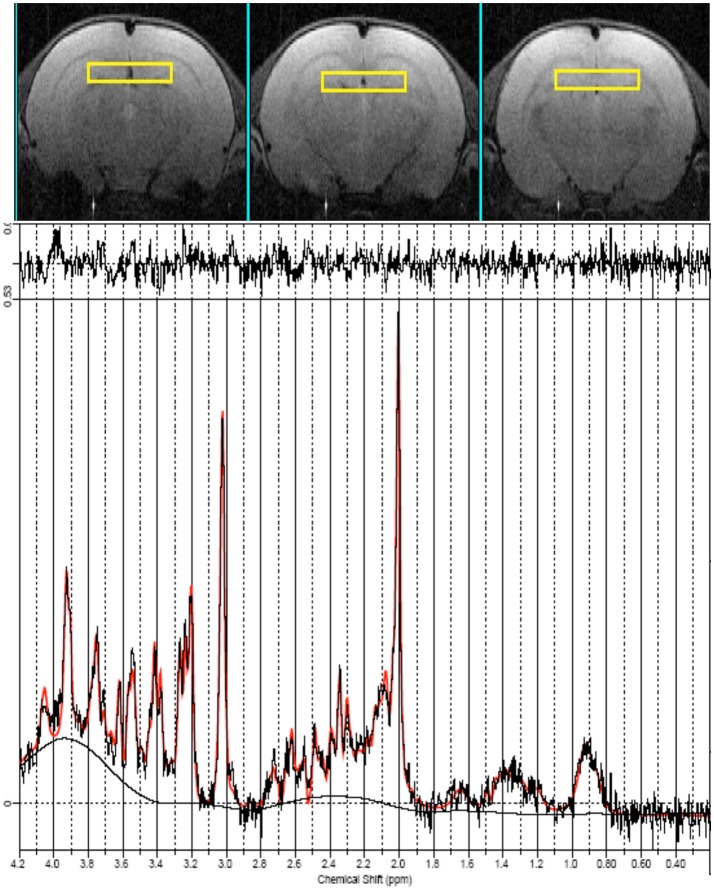
MRI panel—Example of *in vivo* axial T2-weighted spin-echo consecutive images (TR/TE = 2,500/50 ms, slice thickness 1 mm, NS = 2, FOV = 25 × 25 mm^2^, matrix 128 × 256) of a “mid” age-stage rat (PND 28–32). The Voxel is localized on the anterior part of the Hip and is indicated by the yellow rectangles. MRS panel—Representative *in vivo* 1H spectra (black trace), acquired from the Hip (PRESS, TR/TE = 4,000/23 ms, NS = 256). Overimposed (red trace), the result of LCModel fit.

Finally, ^1^H localized MR spectra (PRESS sequence, TR/TE = 4,000/23, ns = 512) were collected at early, mid and late age stages from the anterior part of the hippocampus which include the dentate gyrus (ROI = 18 μl, shown in Figure 3 on the axial images as yellow squares) according to a quantitative protocol (Adriani et al., 2007; Marco et al., 2007; Canese et al., 2012). Briefly, long repetition time was selected, in order to minimize the potential bias due to the spin-lattice (T1) relaxation times. Water T2 measurement was performed at each age and the quantitative metabolite levels were corrected according to it. The unsuppressed water signal acquired from the same voxel was used as an internal reference for metabolite quantification and assuming water content of 81% (Schwab et al., 1997). In this way, we were confident to attribute the observed changes in signal intensities to actual changes in metabolite levels during adolescence. Field homogeneity was optimized by manual shimming (first and second order) up to signal line widths ranging between 5 and 9 Hz for water signal. Spectral metabolic profiles were acquired under conditions of efficient water signal suppression, by VAPOR technique pre-sequence composed by seven CHESS pulses with optimized flip angles and timing in order to have a reduced sensitivity to B1 variation (thus, it is highly efficient also for surface coil). The number of scans was sufficient to obtain good sensitivity spectra from such a small voxel in 34 min.

The spectral region between 0.2 and 4.0 ppm of *in vivo* MR spectra was analyzed by using the LCModel 6.1 fitting routine (Provencher, 1993). The LCModel analysis calculates the best fits to the experimental spectrum as a linear combination of solution spectra of brain metabolites.

Seventeen metabolites were included in the basis set: alanine (Ala), aspartate (Asp), creatine (Cr), phosphor-creatine (PCr), γ-aminobutyric acid (GABA), glucose (Glc), glutamate (Glu), glutamine (Gln), glycerol-phosphoryl-choline (GPC), guanido-acetate (Gua), phospho-choline (PCho), myo-inositol (Ins), lactate (Lac), N-acetyl-aspartate (NAA), N-acetyl-aspartyl-glutamate (NAAG), scyllo-inositol, and taurine (Tau). Spectra of lipids and macromolecules were also included in the basis set. We included in the analysis only those metabolites that were estimated to have Cramer-Rao lower bounds (CRLB) less than 20% (which corresponded to an estimated concentration error <0.2 μmol/g). For signals arising from two or more overlapping resonances, the fitting program calculates the best fit for both the entire resonance (for example: Glx = Gln + Glu) and the separate contributions (Gln and Glu). Metabolite concentrations are expressed in mmol/liter (mM) and significant differences values between the groups were assessed by one-way ANOVA. An example of LCmodel analysis is shown in Figure 3.

## Results

The heart rate, pulse distension, and breath rate were measured during the resting state fMRI session. The initial 4 min and the final 4 min, of the resting state fMRI study, are summarized in Table 1. No statistical differences were found in any physiological parameter among the three ages (one-way ANOVA), neither pre nor post functional acquisitions.

Differences in functional connectivity among key forebrain networks were investigated during the development. Increased strength of connectivity was detected between early and mid ages (juvenile transition) as well as between mid and late ages (adolescence transition) for all analyzed seeds. For direction of connectivity, we considered that voxels passing the threshold have a small volume (0.4 × 0.4 mm) while seeds, being composed of two/three × two/three voxels, have a four- to-nine-fold volume: it is therefore likely that neuronal bodies, which lie inside voxels, are identified when the seed is placed onto their dendritic tree, rather than the other way round (see also Zoratto et al., 2017). Such notion deserves obviously further histological confirmation.

To recapitulate information contained in the extracts of network changes between early and mid ages (juvenile transition in rats), it's interesting to notice that the only pattern of enhanced connectivity which is both bilateral and specular is the one that from both Striata reaches Hippocampi of both hemispheres. In only 1 week, one can observe bilateral growth in connectivity from left Cerebellum to both Orbitofrontal Cortex, from Midbrain to both Hippocampi and from there to Nucleus accumbens and Orbitofrontal Cortex, both belonging to left hemisphere. Other changes highlighted by data, that are neither bilateral nor specular, involve a number of regions: enhanced connectivity can be observed from Cerebellum to Hippocampus both belonging to the right hemisphere, from left Thalamus to ipsilateral Hippocampus, from right Striatum and Orbitofrontal Cortex to ipsilateral Nucleus accumbens, and vice versa from there to ipsilateral Orbitofrontal cortex, from left Prefrontal Cortex to ipsilateral Orbitofrontal Cortex and eventually from left Striatum, Nucleus accumbens, Insular and Prefrontal Cortex to contralateral Orbitofrontal Cortex (see example of Figure 1).

After 1 week, we noticed further changes in connectivity surviving in the comparison between late- and early- age-stage brains, once removed those already emerging for mid- vs. early- age-stage brains. Namely, it's particularly relevant the fact that connections between Hippocampi and Striata of both hemispheres, already seen in the previous week, keep on growing. During this period, other patterns which are both bilateral and specular appear, with arrows from Cerebellum and Hyphothalamus to Prefrontal Cortex and from Thalamus to Striatum. We can also observe patterns of enhanced connectivity which are specular but not bilateral: Striata of both hemispheres are reached from ipsilateral Prefrontal Cortex and contralateral Frontal (motor) Cortex, both Orbitofrontal Cortex from ipsilateral Superior Colliculus, both Nucleuses accumbens from contralateral Striatum and finally both Hippocampi from ipsilateral Hippocampus. There are also several patterns that are only bilateral: from both Amygdalae to right Hippocampus, from left Internal Capsule to both Prefrontal Cortex, from both Hippocampi to right Hippocampus and from both Internal Capsule to right Orbitofrontal Cortex. Finally, we can mention those patterns which are neither bilateral nor specular, where arrows connect right Midbrain and Hippocampus with contralateral Nucleus accumbens and right Thalamus with contralateral Hippocampus.

Some of these connections are of particular interest, because regions involved in the process may explain some typical behaviors that occurs and develop during adolescence, such as increased social behavior (Csikszentmihalyi et al., 1977), novelty and sensation seeking (Adriani et al., 1998; Stansfield et al., 2004; Stansfield and Kirstein, 2006), tendencies toward risk taking (Spear, 2000; Steinberg, 2008; Doremus-Fitzwater and Spear, 2016), emotional instability (Steinberg, 2005) and impulsivity (Fairbanks et al., 2001; Adriani and Laviola, 2003; Chambers et al., 2003; Vaidya et al., 2004; Doremus-Fitzwater et al., 2012). Independently from the age when these connections develop, Striata of both hemispheres send axons to both Hippocampi and Nucleuses accumbens, both Hippocampi reach left Nucleus accumbens and Orbitofrontal cortex and finally both Nucleuses accumbens reach right Orbitofrontal cortex.

DTI data shows a significant decrease of FA in the brains of animals at late compared to mid age (adolescence transition) in both NAcc (*p* = 0.042) and OFC (*p* = 0.016), with a slight (but not significant) reduction of MD, as shown in Figure 2D. The FA decrease is due to a reduction in both axial and radial diffusivity in those regions (not shown). No significant differences neither for FA nor for MD were detected for Hippocampus, PFC and Striatum.

**Figure 4:**
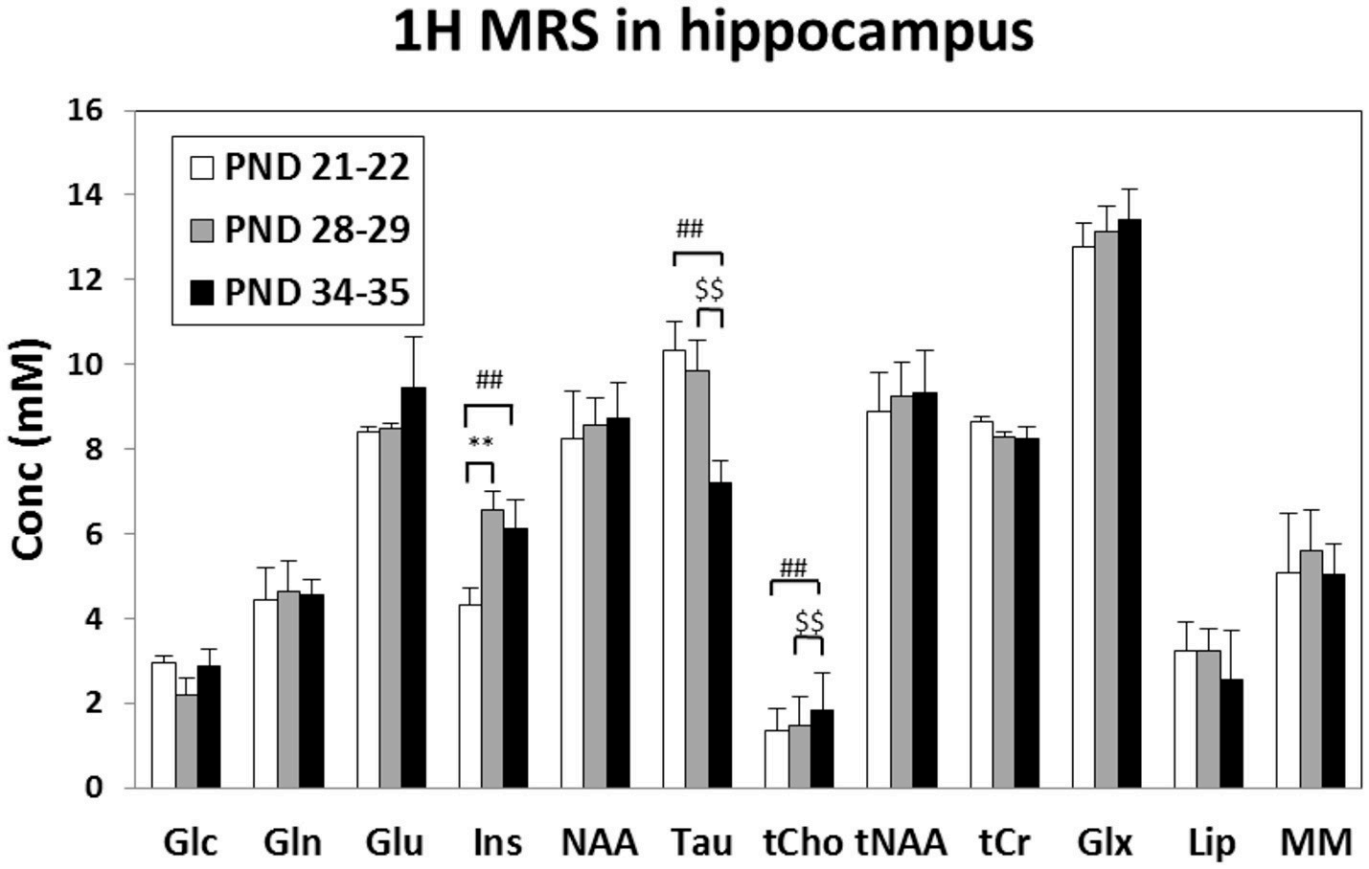
Metabolite concentrations (mM), corrected for water T2 and referred to water signal, measured in the rat Hip at “early” (PND 21–25), “mid” (PND 28–32) and “late” (PND 35–39) age stages in rats. ^**^Early vs. mid *p* < 0.01 (juvenile transition); ^##^early vs. late *p* < 0.01; ^$$^mid vs. late *p* < 0.01 (adolescent transitions). Metabolite assignments: Glc, glucose; Gln, glutamine; Glu, glutamate; Ins, inositol; NAA, N-acetyl-aspartate; Tau, taurine; tCho, total choline (phospho-choline plus glicero-phospho-choline); tNAA, total of NAA plus N-acetyl-aspartyl-glutamate; tCr, total creatine; Glx, total Gln+Glu; Lip, lipids at 1.3 ppm; MM, macromolecules.

Although no change was observed during juvenile transition (mid vs. early), a significant trend suggested a decrease of T2 in adolescence transition (mid, 68.2 ms vs. late, 63.7 ms, *p* = 0.056); therefore, a reduced water molecule tumbling due to increased interactions with macromolecules was likely observed. Quantitative MRS data analysis (see Figure 4) detected an increase of Ins and Cr in mid vs. early ages (juvenile transition). We also detected an increase in total choline and a decrease of Tau in late vs. mid ages (adolescence transition).

## Discussion

To date, these are the first observations in rodents, which explore functional, structural and metabolic modifications during transitions into juvenile age and then adolescence. This is a crucial time window for brain development (Spear, 2000; Laviola et al., 2003): by studying brains with functional MRI and MRS at three distinct developmental periods, we aimed at characterizing two major transitions, namely the juvenile (“mid” vs. “early”) and the adolescent (“late” vs. “mid”) one. We describe in the present paper how patterns of brain connectivity change during adolescent rat development: however, what the emergence of each particular connection means from a functional viewpoint is obviously less easy to explain. The importance of neural connectivity, which links brain regions to each other (Paus, 2005), resides on the fact that different areas of the mammalian brain are specialized in processing different types of information. Nevertheless, coherently with information contained in the diagrams, what function is directly exerted by enhanced connections within the brain is less immediate to understand. We may hypothesize that the growth and establishment of a particular connection is probably associated with the emergence of behaviors that are typical of adolescence. The daily evolving of brain's structure and its developmental discontinuities during infancy exert an influence directly on rodent behavior (Brenhouse and Andersen, 2011).

The period between 21 and 38 PND in rats, which roughly corresponds to 9–14 years of age in humans (Laviola et al., 2003), is characterized by synaptic overproduction (Brenhouse and Andersen, 2011), followed by a phase with loss of synapses (“pruning”) that occurs afterwards (Chechik et al., 1999). In the mentioned age range, with the establishment of circuits that are sometimes redundant and not so functional as well, connectivity becomes progressively more complex (Marco et al., 2011): in general terms, subcortical and posterior regions start to communicate mainly with cortical and anterior areas: the consequence is a changing balance between limbic/subcortical and frontal lobe functions (Giedd, 2008). Classically, the latter are slower to mature than the former ones: for instance, during adolescence the Nucleus accumbens' response to reward is greater than the Orbitofrontal cortex's one (Ernst et al., 2005; Galvan et al., 2006): such developmental mismatch is thought to explain why adolescents' reward process differs from adults (Sturman and Moghaddam, 2011). However, while development proceeds, networks become more and more distributed as different regions become more interconnected (Fair et al., 2009), with the result of improving functional coordination between different brain structures.

**Figure 5:**
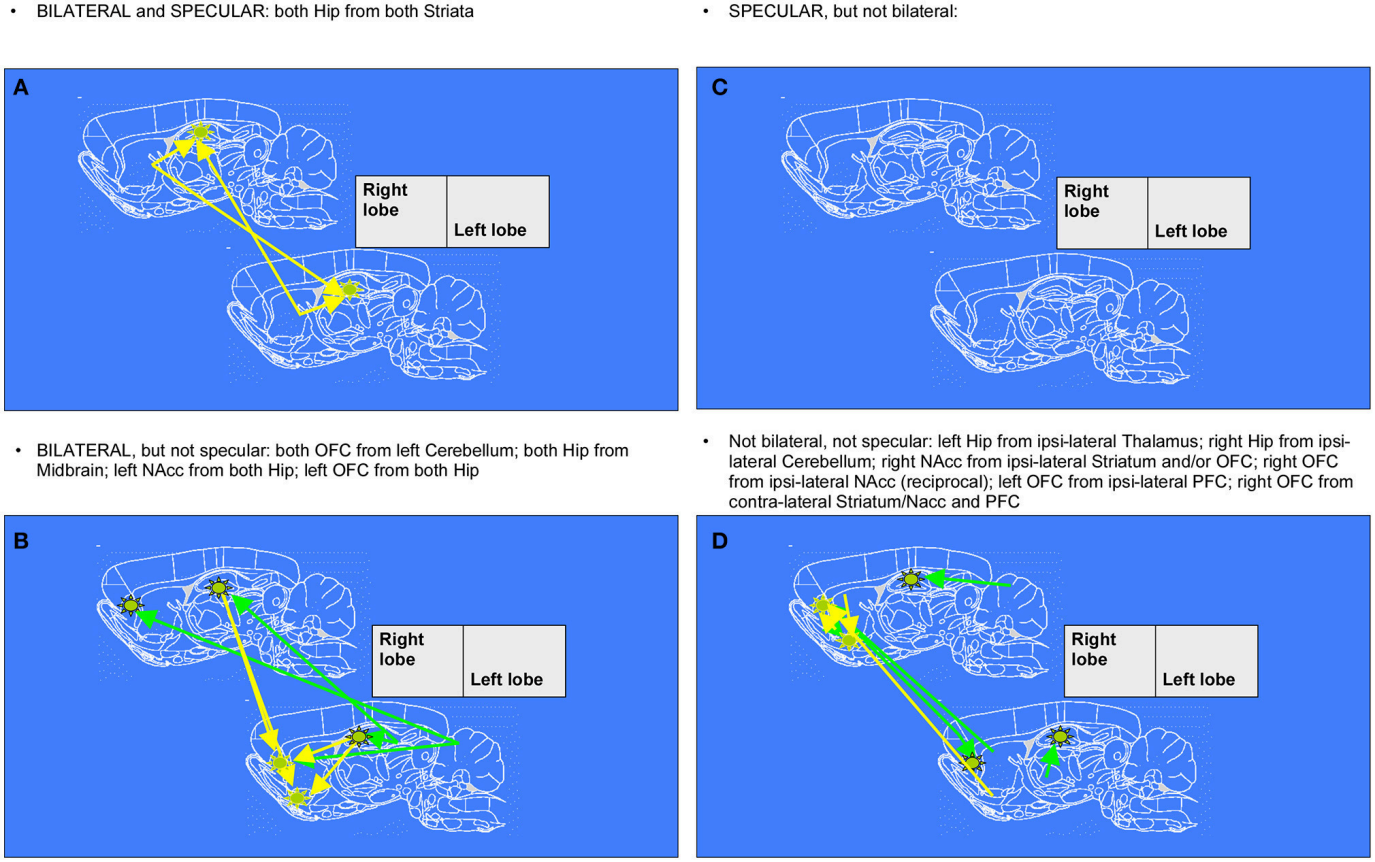
Diagrams denoting increased connections between “early” and “mid” age stages in rat brain (juvenile transition). Rat brain atlas diagrams (Paxinos and Watson, 1998), corresponding to the center of the MRI slices, are used to plot arrows denoting connections to a seed (see stars) from “target” regions where pixels consistently emerge. This happens when the cross-correlation value in the template for “mid” age stage group is trespassing the corresponding threshold value (for the same pixel) calculated from resampled “early” age stage group (see Figure 1 for an example of difference-from-threshold maps). Arrows denote the regions whose connectivity to that seed (see stars) was enhanced (higher than the 99th percentile of the resampled “early” age stage). (**A,C**) specular changes (**B,D**) not specular changes (**A,B**) bilateral changes (**C,D**) not bilateral changes (see definitions in Figure 6).

**Figure 6:**
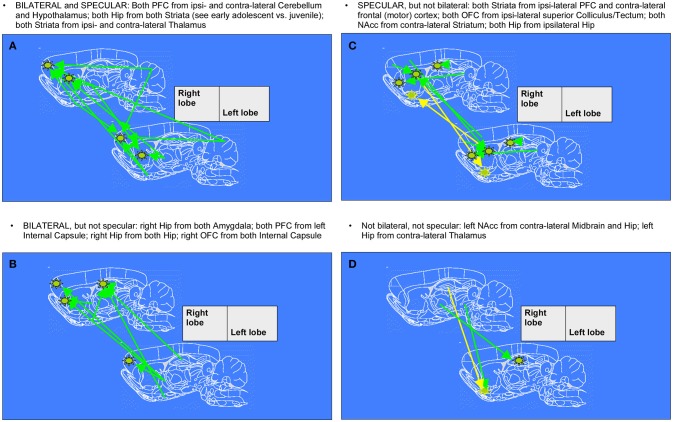
Diagrams denoting increased connections between “early” and “late” age stages in rat brain (adolescent transition). The meaning of the arrows is the same as in Figure 5. (**A,C**) specular changes (**B,D**) not specular changes (**A,B**) bilateral changes (**C,D**) not bilateral changes. The “specular” situation is that in which a same-seed to same-region connection emerges as mirrored (i.e., fully corresponding pixels, but on the other hemisphere) when a seed is placed both on left lobe and on right lobe (see **A,C**). The “bilateral” situation is that in which, when seeds are placed on both sides of area A, a connection coming not from the “mirror” but from the “very same” region (i.e., identical or neighboring pixels) emerges, thus appearing first ipsi-laterally and then contra-laterally (see **A,B**).

A quick look to our data, summarized by the diagrams (Figures 5, 6), suggests that some patterns of enhanced connectivity continually develop during the period we considered: in particular, what strikes most at a glance are those arrows that connect the Hippocampus from the Striatum. These two regions are highly involved in the two major memory systems: the explicit (or declarative) memory system, also including spatial memory, which is under the control of the Hippocampus, and an implicit (or procedural) memory system, depending on habit-based functions exerted by the Striatum (Goodman and McIntyre, 2017). Enhanced connections between these two structures may entail a major integration between the two systems depicted above, and can likely explain a better performance in some specific tasks, such as reversal learning and inhibitory avoidance learning (Rossato et al., 2006). The maturation of such a set of skills allows individuals that are approaching adulthood to reach independence from parental care (Laviola et al., 2003).

Another pathway highlighted by our data is the one that, from the Striatum, reaches the Nucleus accumbens. Together, these two nearby structures have a critical role in habit formation and/or exploitation and behavioral flexibility respectively (Ragozzino, 2003; Yin et al., 2004): as mentioned above, the dorsal Striatum is involved in action selection but also in association learning (Sturman and Moghaddam, 2011), while the Nucleus accumbens, a major target of the meso-limbic accumbal pathway, subserves motivation involving the release of dopamine (DA) (Grace et al., 2007). During adolescence, a reduced DA release in basal conditions and a greater release, which occurs after stimulation due to a larger DA storage pool when compared to adults, may explain the lower basal levels of motivation and boredom typical of this age (Laviola et al., 2003). Very old and classical studies depict the psychopharmacological consequence of meso-limbic development: at the onset of adolescence, rodents are hypo-responsive to amphetamine and express explorative investigation (Lanier and Isaacson, 1977; Shalaby and Spear, 1980; Spear and Brake, 1983), (Adriani et al., 1998); also, learning abilities before puberty are solely based on the simpler psychomotor responses not requiring an integration with cognitive and attentional skills (Newman and McGaughy, 2011). This profile is due to a transient age-related reduction of DA release in basal condition, a notion confirmed recently with dopamine transporter knock-out rats (Adinolfi et al., 2018). Highly consistently, adolescent rodents appear more impulsive and unable to perceive the rewarding power of amphetamine (Adriani and Laviola, 2003).

Notably, both Striatum and Nucleus accumbens are involved in association learning, but with different roles: the latter adjusts the strength of conditioning by modulating the link between stimuli and appetitive or aversive reactions (Horvitz, 2002) and is critical for motivated learning of performance; on the other hand, the Striatum, which plays a role in stimulus-response (S-R) habit-learning (Yin et al., 2008), is involved in performance, but not in learning (Atallah et al., 2007). Following that line of reasoning, enhanced connections between them may assure a better integration of their complementary functions during transition from adolescence into adulthood: the likely consequence is a progressive fading of the tendency to take risks and to get involved in dangerous situations with better performances in operant choice and instrumental conditioning tasks (Adriani and Laviola, 2003; Hunt et al., 2016).

The Nucleus accumbens also represents a site of integration for limbic information processed by the Hippocampus and the Amygdala (Mogenson et al., 1980). The latter region is known for processing the associations between an external stimulus and emotional reactions, mostly fear (McDonald and White, 1993; Squire et al., 1993; Aggleton, 2000). These three regions, which altogether constitute a limbic network involved in affective processing (Burnett et al., 2010), during the age period we considered show a pattern of enhanced connectivity, indicative of maturing projections between each other: from the Amygdala to Hippocampus and then, in a more remarkable way, from the Hippocampus to Nucleus accumbens. The latter, especially in its shell, plays a role in the appetitive spatial performance by translating spatial information processed by the Hippocampus into action (Ito et al., 2008). This latter, in turn, is modulated by events that are emotionally meaningful (Kahn and Shohamy, 2013). These regions are highly involved in a neural pathway fundamental for the individual in order to show adaptive skills: since an emotion is felt, a memory for that place is formed and later a drive toward (or away from) that place is originated. The development of these limbic connections probably improves, during transition from adolescence into adulthood, the contextual-dependent memory retrieval and place learning: accordingly, amphetamine-induced place conditioning is dampened while simple psychomotor sensitization is enhanced in adolescent mice (Adriani and Laviola, 2003; Tirelli et al., 2003).

In the comparison between early and mid age-stage (juvenile transition in rats) data, both Nucleus accumbens and Hippocampus undergo maturation of functional enhanced projections to the Orbitofrontal cortex. This last structure, corresponding to the most inferior and ventral parts of Prefrontal cortex, is involved in a number of functions, including the reappraisal of the link between stimulus and reward (Happaney et al., 2004). For what concerns the Nucleus accumbens, the projections connecting it to the Orbitofrontal cortex are reciprocal and particularly interesting: these two structures together constitute a keynote circuit, implied in some important functions. Namely, by gathering information from the Nucleus accumbens, implicated in associations between stimulus and reward, the Orbitofrontal cortex accounts for the affective evaluation of outcome features and the feedback modulation of future choice (Stott and Redish, 2014): derived from NAcc-to-OFC processing is a signal that encodes the “subjective reward value” compared to own expectation and guiding anticipation of probable payoff (Canese et al., 2011). Indeed, it has been observed how adolescents show an exaggerated Nucleus accumbens response relative to children in reward-seeking behavior, similarly to adults, while the Orbitofrontal cortex undergoes a more diffuse recruitment, more similar to children than to adults (Galvan et al., 2006). We previously mentioned how the development mismatch between these two structures explains adolescents' tendencies to take risks and make impulsive choices leading to addiction-related problems (Laviola et al., 2003).

Functional interactions between the Hippocampus and the Orbitofrontal cortex appear to be critical in order to guide appropriate social behavior. This circuitry seems to be implied in creating, maintaining and retrieving overlapping representations of another individual by monitoring the facial expressions of such individual in working memory (Ross et al., 2013). Deciphering and making inferences about others' mental state is a typically human feature known as “theory of mind” (Stone et al., 1998), but other mammals and rodent as well can present empathy-like skills (Ben-Ami Bartal et al., 2011) which also act as an emotional regulator (Burnett et al., 2010). An improvement of coordination between hippocampal and orbitofrontal areas, which occurs early during adolescent development, may support social interactions with peers (in humans, or with conspecifics in mammals), which are of primary importance during adolescence. Another structure implied in some complex cognitive functions, such as attention and short term memory (Rolls, 2008), is the medial Prefrontal cortex, one of the last region to mature (Gogtay et al., 2004). During the period considered in the present study, a pattern of enhanced projections from this region to the Orbitofrontal cortex can be seen. Indeed, these two regions interact with the same limbic circuitry; notably, the information encoded by both the (ventral) Orbital and the (dorso) medial portions of PFC is then used for decision making (Canese et al., 2011).

The patterns of enhanced connectivity depicted above and summarized in Table 2 show that the Striatum indirectly reaches the Orbitofrontal cortex passing through the Hippocampus and the Nucleus accumbens (with the latter also reaching the former). It's well-known that the Orbitofrontal cortex, together with the dorsal and ventral Striatum, belongs to a circuitry involved in the establishment of the “subjective reward value” by predicting and then monitoring the outcome of any action and any choice (Padoa-Schioppa and Assad, 2006, 2008; Kable and Glimcher, 2007; Lau and Glimcher, 2008; Plassmann et al., 2010). The Hippocampus probably interacts with these regions in order to make a decision: its role may be to help in those circumstances when specific reward value, associated with the predicted outcome of an option, can be better implemented by considering the current spatial context where that decision has to be made (Ross et al., 2011). According to our data, summarized in the matrix of Table 2 and in the diagrams of Figures 5,6, the adolescent establishment of enhanced projections is directional and can be observed in only one way: from the Striatum to the Orbitofrontal cortex. Of note, the latter plays a role in reversal learning, which is the action revaluation and consequent ability to adapt behavior after a negative feedback (Fineberg et al., 2010). Moreover, the dorsal Striatum can be divided into two different portions, each of them playing a different role: while the dorsal lateral Striatum (DLS) is involved in habits, the dorsal medial Striatum (DMS) is necessary for goal-directed behavior (Yin et al., 2004, 2005). It seems that our data identify cortico-basal ganglia network centered on the dorsomedial striatum, subserving the acquisition of goal-directed actions (Hart et al., 2014). Enhanced projections from striatal to orbitofrontal areas may assure cognitive flexibility and prevent both compulsive and impulsive behavior: both may be poorly adaptive for an individual who has left the nest behind and is now approaching the world. Future experiments are warranted to explain why impulsiveness is so frequently associated with adolescence: animal models could well try to reproduce those altered trajectories and developmental discontinuities which may interest connections among limbic and cortical structures that see the latter to develop functional control over the former.

**Table 2:** Summary of the results depicted in Figures 5, 6 (Note: contents follow the format side of target → side of seed and shall be read as “from” target “to” seed).

**Target →** **↓Seed**	**Hippocampus**		**Nucleus accumbens**		**Orbitofrontal cortex**		**Prefrontal cortex**		**Striatum**	
	**Ipsilateral**	**Contralateral**	**Ipsilateral**	**Contralateral**	**Ipsilateral**	**Contralateral**	**Ipsilateral**	**Contralateral**	**Ipsilateral**	**Contralateral**
	R → R	R → L	R → R	R → L	R → R	R → L	R → R	R → L	R → R	R → L
**From Right-sided target**	ADOLESCENT								JUVENILE & ADOLESCENT	JUVENILE & ADOLESCENT
**Hippocampus**	L → L	L → R	L → L	L → R	L → L	L → R	L → L	L → R	L → L	L → R
**From Left-sided target**	ADOLESCENT	ADOLESCENT							JUVENILE & ADOLESCENT	JUVENILE & ADOLESCENT
	R → R	R → L	R → R	R → L	R → R	R → L	R → R	R → L	R → R	R → L
**From Right-sided target**		JUVENILE & ADOLESCENT			JUVENILE				JUVENILE	ADOLESCENT
**Nucleus accumbens**	L → L	L → R	L → L	L → R	L → L	L → R	L → L	L → R	L → L	L → R
**From Left-sided target**	JUVENILE									ADOLESCENT
	R → R	R → L	R → R	R → L	R → R	R → L	R → R	R → L	R → R	R → L
**From Right-sided target**		JUVENILE	JUVENILE							
**Orbitofrontal cortex**	L → L	L → R	L → L	L → R	L → L	L → R	L → L	L → R	L → L	L → R
**From Left-sided target**	JUVENILE			JUVENILE			JUVENILE	JUVENILE		JUVENILE
	R → R	R → L	R → R	R → L	R → R	R → L	R → R	R → L	R → R	R → L
**From Right-sided target**										
**Prefrontal cortex**	L → L	L → R	L → L	L → R	L → L	L → R	L → L	L → R	L → L	L → R
**From Left-sided target**										
	R → R	R → L	R → R	R → L	R → R	R → L	R → R	R → L	R → R	R → L
**From Right-sided target**							ADOLESCENT			
**Striatum**	L → L	L → R	L → L	L → R	L → L	L → R	L → L	L → R	L → L	L → R
**From Left-sided target**							ADOLESCENT			

*On separate rows, we report the location of seeds; on separate columns, we report the location of ‘target’ areas, namely the active voxels identified by the software as trespassing the threshold of the 99% percentile from the re-sampled distribution of “control” subjects at the post-weaning age-stage (see section Materials and Methods). Each cell in the Table then reports about ipsi-lateral (R → R and L → L) or contra-lateral (R → L and L → R) change (R, right; L, left; “ → ” denotes the direction; the connection proceeds from the target to the seed). “Juvenile” transition means that the targets with active voxels are found in the analysis for “mid” age-stage; “Adolescent” transition means that the targets with active voxels are found for “late” age-stage; if both are mentioned, the value at “late” age-stage is further increased compared to what evident at “mid” stage*.

Diffusion tensor imaging (DTI) studies have provided much evidence of white and gray matter alterations during childhood and adolescence in humans (Tamnes et al., 2018) that suggest increasing myelination, axon density, and/or fiber coherence. White matter development has been extensively investigated by DTI, while recent studies are starting to explore gray matter with DTI techniques (Mah et al., 2017). The age-related increases of FA and decreases of MD were observed between childhood and adolescence but no study has been performed so far in rodents. Thus, we have been the first (to our knowledge) to use MRI and MRS to distinguish amongst different periods within adolescence. Our studies suggest that changes within subcortical gray matter, as in white matter, can be observed in NAcc and OFC at the late age stage (i.e., full adolescence) and could be due to increasing neurite density rather than changes in neuronal geometry (Mah et al., 2017). These results may be consistent with the important role exerted by these two structures in adolescent behavior previously highlighted by fMRI data. Intriguingly, this structural change seems to follow the mentioned maturation of functional, and noteworthy reciprocal (at least on the right hemisphere), connections between NAcc and OFC, occurring between early and mid age stages (juvenile transition in rats). It is tempting to speculate that this may be a common pattern: regions may first reach a reciprocal functional inter-connection (evidenced by rs-fMRI) and subsequently undergo a series of structural changes (evidenced by DTI) which are consistent with increased number of neurites and/or decreased number of neuronal cell bodies. It is noteworthy that all these changes involve the tight inter-connection between NAcc and OFC which, together, are well-known to subserve reward processes like estimation and prediction of reward values, anticipation and detection of salient contingencies (Padoa-Schioppa and Assad, 2006, 2008).

The levels of metabolites observed in the hippocampus are in agreement with previous works which analyzed the rat hippocampus during early infant development (from p7 to p28) (Tkáč et al., 2003) or across the entire developmental period (from p10 to p70) (Ramu et al., 2016). To date, no one has investigated on the difference in the metabolism during the specific transitions (the juvenile and the adolescence one) occurring in this period. Firstly, we observed a consistent increase in myo-inositol, which is a marker of glial cells. This, suggesting an increase in glial processes during passage between early and mid age stages (juvenile rather than adolescence transition).

Taurine reduction at the “late” age period suggests that the initial part of adolescence could be characterized by reduction in cell proliferation, because of the known role of taurine in neural stem/progenitor cell proliferation in developing brain (Shivaraj et al., 2012). Taurine is also associated to the levels of proteins associated with synapse development: therefore, its reduction is consistent with “pruning” (Spear, 2000; Blüml et al., 2013).

Choline is an essential nutrient which showed neuroprotective effects in both animal and human studies (Blusztajn et al., 2017). The synaptogenesis, a process that occurs throughout life, is enhanced by choline supplementation. Moreover, elevated acetylcholine synthesis and release from forebrain cholinergic neurons (required for normal cognitive and memory function), were associated with synaptogenesis (Wurtman, 2014). The increased levels of total choline suggest an increase of activity of cholinergic neurons in the “late” age, consistent with an enhanced synaptogenesis (in those specific neurons surviving from pruning processes).

Thus, interestingly, taurine reduction as well as total choline increase take place in close timing with adolescent transition, when posterior and anterior brain regions, as well as subcortical and cortical areas, become more and more interconnected, promoting the establishment of a complex and coordinated brain circuitry.

## Conclusions

In this work, we adopted an anesthesia protocol already validated in our previous connectivity studies (Canese et al., 2015; Zoratto et al., 2017), where resting-state BOLD signal fluctuations were reliably detected. The 2% isoflurane was necessary to maintain very young rats under a stable and prolonged anesthesia, with minimal physiological alterations. Lower levels of isoflurane are not sufficient to keep very young animals in proper conditions which allow the acquisition of BOLD images with accuracy and quality. It was shown that BOLD signals under a 2% isoflurane are similar, but lower, than in awake animals (Liang et al., 2013); our BOLD signals could still be somewhat reduced when compared to a lower level of isoflurane anesthesia: we therefore likely detect only the stronger correlations between the seed and brain areas.

Present data provide a deeper view on the metabolic, morphological and functional modifications which occur during rodents' adolescence. Our MRS findings include reduction of taurine and increase in total choline, which can be considered respectively as an index of neuronal pruning and of synaptic efficiency (in non-pruned neuronal cells). This, noteworthy, was evidenced in the Hippocampus from mid to late age stages (adolescence transition). The FA reduction suggest that important structural modifications happen in the NAcc and OFC during adolescence transition. Clearly, this is in agreement with evidenced maturation of reciprocal connection between these two areas. Finally, it is during adolescence transition that functional changes occur within forebrain networks: a maturation of hippocampal-striatal connection and circuitry of the limbic loops was clearly evidenced.

This is an inaugural paper, therefore further studies of this kind may well follow. For instance, a more detailed account of further changes occurring following the onset of puberty is warranted: the period between the onset of puberty (PND 40) and reach of adulthood (classically, PND > 60) may well be investigated in the same way, taking into account the seventh (PND 42), eighth (PND 49) and ninth (PND 54) week of life. A future study will certainly address if female gender differs from the male one in terms of timing and nature of the maturing connections. In another perspective, the loss of connectivity during aging is certainly deserving investigation (Ash et al., 2016): this could be realized by comparing brains at 2 months of age with those at 6, 12, 18 months, and perhaps older.

## Authors contributions

WA, RC, and GL designed the experiments. FZ and LA carried out the experiments. LA, NT, and RC analyzed the data. NT, WA, and RC wrote the manuscript. GL contributed with specific expertise.

### Conflict of interest statement

The authors declare that the research was conducted in the absence of any commercial or financial relationships that could be construed as a potential conflict of interest.
